# Prevalence of Darunavir Resistance in the United States from 2010 to 2017

**DOI:** 10.1089/aid.2018.0100

**Published:** 2018-12-11

**Authors:** Kimberley Brown, Lisa Stewart, Jeannette M. Whitcomb, Dongmei Yang, Richard E. Nettles, Erkki Lathouwers

**Affiliations:** ^1^ Janssen Scientific Affairs, LLC, Titusville, New Jersey.; ^2^ Monogram Biosciences, LabCorp, South San Francisco, California.; ^3^ Janssen Infectious Diseases BVBA, Beerse, Belgium.

**Keywords:** darunavir once daily, human immunodeficiency virus-1, genotypic resistance, resistance-associated mutations, phenotypic resistance, antiretroviral

## Abstract

The emergence and transmission of antiretroviral drug resistance have been and remain a concern among people living with human immunodeficiency virus (HIV)-1 infection. The protease inhibitor (PI) darunavir has been approved for use in the United States for more than 10 years and has demonstrated a high barrier to resistance. Previous analyses identified significant reductions in the prevalence of samples with darunavir resistance-associated mutations (RAMs) and with phenotypic resistance to darunavir and other PIs between 2006 and 2012. This analysis extends those findings by evaluating darunavir and PI resistance among clinical samples submitted for routine drug resistance testing (combined genotyping and phenotyping) in the United States from 2010 to 2017. Frequencies of 11 darunavir and 23 primary PI RAMs, and phenotypic susceptibility, were assessed yearly among all samples and in a subset of samples with distinct phenotypic resistance to one or more PIs. Among all samples (*N* = 60,760), the proportion with 0 darunavir RAMs was 91.7% in 2010 and 95.8% in 2017. The proportions of all samples with phenotypic susceptibility to darunavir, atazanavir, and lopinavir were, respectively, 97.4%, 94.2%, and 94.7% in 2010 and 98.6%, 97.7%, and 97.5% in 2017. Among the 4,799 samples with phenotypic resistance to one or more PIs, the proportions with phenotypic susceptibility to darunavir, atazanavir, and lopinavir were, respectively, 73.3%, 41.5%, and 46.0% in 2010 and 70.7%, 53.7%, and 48.8% in 2017. The prevalence of darunavir RAMs among commercially tested HIV-1 samples remained low and generally stable from 2010 to 2017, and high proportions showed phenotypic darunavir susceptibility.

## Introduction

The emergence and transmission of antiretroviral (ARV) drug resistance have been and remain a concern for people living with human immunodeficiency virus (HIV)-1 infection.^1,2^ The resistance profile of the virus and the individual's propensity to be suboptimally adherent to his/her ARV regimen, which ultimately leads to reduced drug levels, are important factors in determining an effective treatment strategy.^1,2^ Another important consideration is the barrier to resistance development of ARV agents, which refers to the ability of the drug to be clinically effective at standard *in vivo* drug levels, despite the presence of viral mutations or suboptimal adherence to therapy.^1,2^ Recent evidence suggests that the overall prevalence of HIV-1 drug resistance has been declining in the United States (US) over time, although the potential for drug resistance remains a concern for certain drug classes.^3,4^ This decline may be due, at least in part, to the availability of simplified treatment regimens with reduced pill burden, leading to better treatment adherence, as well as the high barrier to ARV resistance and improved tolerability of newer ARV agents.^1,3,5–7^ Nevertheless, some individuals with resistance to ARV agents have acquired it as a result of pre-existing resistance-associated mutations (RAMs) in their acquired viral strain or subsequently developed resistance as a result of suboptimal drug levels (due to poor adherence and/or drug potency).^1,3^ Regardless of the cause of the resistance, once obtained, the resistant virus is archived and may reemerge to replicate in the presence of ARVs.

The protease inhibitor (PI) darunavir was initially approved in the US in 2006 for twice-daily dosing (boosted by ritonavir) in treatment-experienced patients, based on an analysis of subjects with triple-class ARV experience with one or more primary PI RAMs (that had been identified at the time).^8^ In 2008, darunavir was approved for once-daily (QD) dosing in treatment-naive individuals and, in 2010, darunavir was approved for QD dosing in treatment-experienced individuals without darunavir RAMs.^9^ Additional darunavir QD dosing formulations have since been developed and include co-formulation with cobicistat (US approval in 2015) and a single-tablet regimen, also containing cobicistat, emtricitabine, and tenofovir alafenamide (D/C/F/TAF; approved in Europe [2017], Canada [2018], and the US [2018]).^10,11^ Darunavir QD boosted by ritonavir or cobicistat and in combination with emtricitabine and tenofovir (including as part of D/C/F/TAF) is recommended for all treatment-naive adults in guidelines from the European AIDS Clinical Society (EACS).^2^ In the US Department of Health and Human Services (DHHS) guidelines, darunavir QD boosted by ritonavir or cobicistat and in combination with tenofovir and emtricitabine (A1 and A2 levels of evidence with each boosting agent, respectively) is recommended as an initial treatment option in certain clinical situations, such as when there are significant concerns about treatment adherence or when resistance test results are unavailable before initiation of ARV therapy (and thus there is a possibility of transmitted resistance).^1^

Eleven HIV-1 protease mutations associated with darunavir resistance have been identified in highly treatment-experienced subjects; notably, the virologic efficacy of darunavir was compromised (i.e., reduced to ≤75% of the overall response rate) in the presence of three or more of these darunavir RAMs in the background of a high number (median of 14 or 15, respectively^12,13^) of International Antiviral Society-USA (IAS-USA) PI RAMs. The high barrier to resistance of darunavir has been demonstrated in numerous studies.^5,12–25^ An analysis of multiple clinical studies of darunavir 800 mg QD-based regimens in treatment-naive and treatment-experienced subjects confirmed that the development of darunavir RAMs and phenotypic resistance was very rare; overall, only 4 of 1,686 (0.2%) subjects had primary PI and/or darunavir RAMs postbaseline, and only one of 1,686 (<0.1%) subjects lost darunavir phenotypic susceptibility (this was possibly related to a prior virologic failure with lopinavir/ritonavir).^5^

A previous study of clinical HIV-1 samples sent for routine resistance testing in the US showed that the prevalence of darunavir RAMs and phenotypic resistance decreased from the time of darunavir approval in 2006 to 2012.^16^ This study is an extension of this prior analysis and was undertaken to provide a current and more robust dataset. In this study, we evaluated darunavir and primary PI resistance mutations and phenotypic resistance patterns to darunavir, as well as the PIs atazanavir (US approval in 2003^26^) and lopinavir (US approval in 2000^27^), observed in clinical samples sent by clinicians for routine drug resistance testing in the US from 2010 to 2017.

## Materials and Methods

### Clinical samples

Clinical samples submitted to Monogram Biosciences (South San Francisco, CA) for routine resistance testing using the PhenoSenseGT^®^ or PhenoSenseGT^®^ plus Integrase assays from January 2010 to December 2017 were evaluated. The dataset was de-duplicated to exclude multiple samples from the same patient within a single calendar year based on collection date; in such cases, only the latest sample was included in the analysis. Patients' ARV treatment experiences were unknown. The PhenoSenseGT and PhenoSenseGT plus Integrase assays are combination resistance tests that provide genotypic detection of RAMs with true phenotypic measurements of drug susceptibility together in the same report. Based on validation experiments, the ability of the assays to detect individual RAMs is dependent on the specific substitution and the background sequence of the individual's HIV-1 strain. The sensitivity to detect variants typically ranges between 10% and 20% of the population.^28^ Phenotypic susceptibility is reported as the fold change in 50% inhibitory concentration (FC-IC_50_), which is calculated as the ratio of the IC_50_ of the sample divided by the IC_50_ of the drug-sensitive reference strain. Validation experiments have demonstrated that reproducibility of replicate phenotypic measurements is typically less than twofold.^29,30^

A subset of samples with phenotypic resistance to one or more PIs, regardless of resistance to other ARV classes, was also evaluated in parallel. Samples were included in this subset if at least one of the PIs evaluated had an FC-IC_50_ of more than two times the lower clinical or biological cutoff for that drug. This provided a stringent selection for samples with phenotypic resistance, excluded samples with minor reductions in susceptibility, and accounted for the inherent variation of phenotypic measurements. The PIs and associated susceptibility cutoffs used to assign samples into the subset of samples with PI resistance were amprenavir (FC-IC_50_ ≥8), atazanavir (FC-IC_50_ ≥10.4), darunavir (FC-IC_50_ ≥20), indinavir (FC-IC_50_ ≥20), lopinavir (FC-IC_50_ ≥18), nelfinavir (FC-IC_50_ ≥7.2), ritonavir (FC-IC_50_ ≥5), saquinavir (FC-IC_50_ ≥4.6), and tipranavir (FC-IC_50_ ≥4).

### Assessment of RAMs

Mutations included in the analysis were based on the IAS-USA protease mutation list.^31^ The 11 darunavir RAMs were V11I, V32I, L33F, I47V, I50V, I54L/M, T74P, L76V, I84V, and L89V. The 23 primary PI RAMs were D30N, V32I, M46I/L, I47A/V, G48V, I50 L/V, I54 L/M, Q58E, T74P, L76V, V82A/F/L/S/T, N83D, I84V, N88S, and L90M.

The proportion of samples with 0, 1, 2, or 3 or more darunavir RAMs; the median number of darunavir RAMs; and the frequency of individual darunavir RAMs were each evaluated at yearly intervals. The median number of primary PI RAMs and the frequency of individual primary PI RAMs were also evaluated at yearly intervals.

### Assessment of phenotypic resistance

The proportion of samples with partial to full phenotypic resistance to darunavir, atazanavir, and lopinavir was evaluated at yearly intervals. Phenotypic resistance was defined by the lower clinical cutoffs for ritonavir-boosted darunavir (FC-IC_50_ >10), ritonavir-boosted atazanavir (FC-IC_50_ >5.2), and ritonavir-boosted lopinavir (FC-IC_50_ >9); boosting with cobicistat was not taken into account. The median darunavir, atazanavir, and lopinavir FC-IC_50_ values were also evaluated at yearly intervals.

### Statistical analysis

The Jonckheere–Terpstra test was performed to analyze the trend significance on yearly data for both all samples and PI-resistant samples. The alternative hypothesis was two sided.

## Results

### All samples

**FIG. 1.**
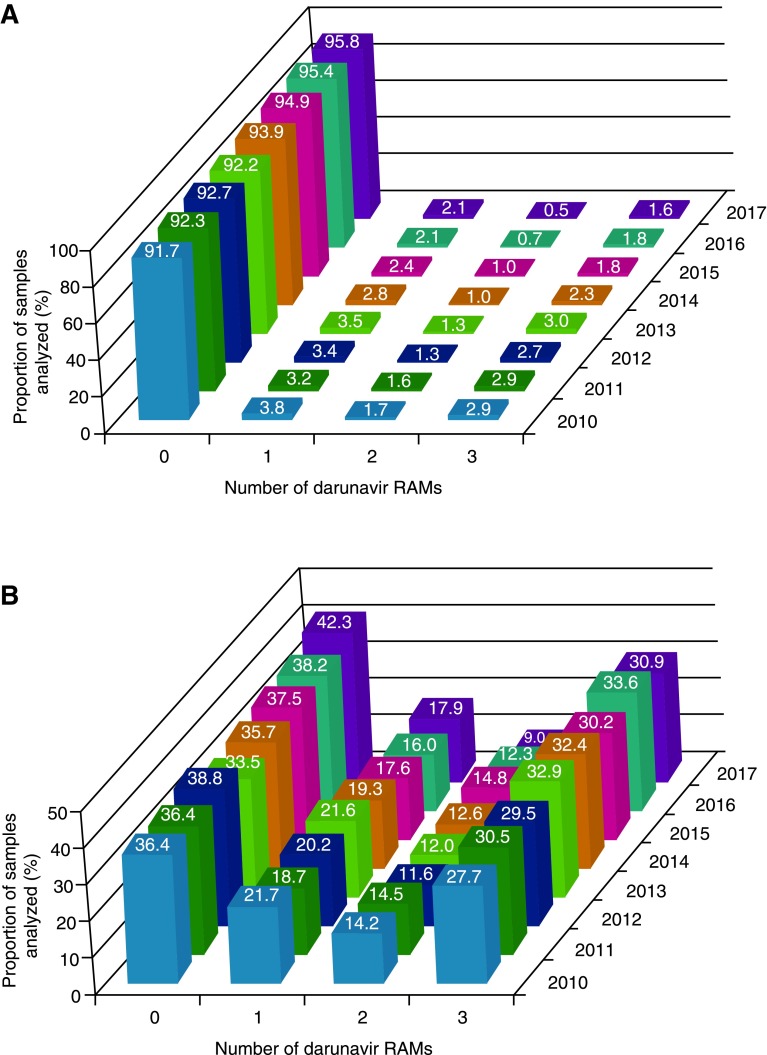
Proportion of samples harboring 0, 1, 2, or 3 or more darunavir RAMs over time for **(A)** all samples and **(B)** samples with phenotypic resistance to one or more PIs. RAM, resistance-associated mutation; PI, protease inhibitor.

**FIG. 2.**
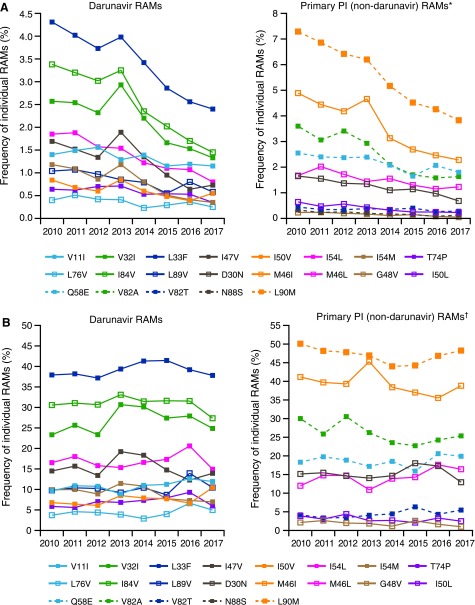
Frequency of individual RAMs over time for **(A)** all samples and **(B)** samples with phenotypic resistance to one or more PIs. RAM, resistance-associated mutation; PI, protease inhibitor. *Only those primary PI (non-darunavir) RAMs that changed in frequency by >0.1% from 2010 to 2017 are shown. Frequency values in 2010 and 2017, respectively, for mutations not shown were as follows: I47A, 0.1% and <0.1%; V82F, 0.1% and 0.1%; V82L, 0.2% and 0.1%; V82S, 0.1% and <0.1%; and N83D, 0.2% and 0.2%. ^†^Only those primary PI (non-darunavir) RAMs that changed in frequency by >1.0% from 2010 to 2017 are shown. Frequency values in 2010 and 2017, respectively, for mutations not shown were as follows: I47A, 0.7% and 0.5%; V82F, 1.4% and 1.0%; V82L, 1.3% and 0.5%; V82S, 0.7% and 0.5%; N83D, 1.3% and 2.0%; and N88S, 1.6% and 2.5%.

A total of 60,760 clinical samples were included in the analysis. Among all samples, the proportion with 0 darunavir RAMs was 91.7% in 2010 and 95.8% in 2017 (*p* = .003), while the proportion with three or more darunavir RAMs was 2.9% in 2010 and 1.6% in 2017 (*p* = .013; Fig. 1A). Correspondingly, the median number of darunavir RAMs was 0 for each year from 2010 to 2017 (*p* = 1.000 [2010 vs. 2017]), and the median number of primary PI RAMs was also 0 for each year during this time period (*p* = 1.000 [2010 vs. 2017]; Table 1). The most common individual darunavir RAMs were L33F (identified in 4.3% of samples in 2010 and 2.4% of samples in 2017; *p* = .001), I84 V (3.4% and 1.5%, respectively; *p* = .003), and V32I (2.6% and 1.3%; *p* = .006); the least common darunavir RAM, L76V, was identified in 0.4% of samples in 2010 and 0.3% of samples in 2017 (*p* = .083; Fig. 2A). The most common primary PI RAM, L90M, was identified in 7.3% of samples in 2010 and 3.8% of samples in 2017 (*p* = .001; Fig. 2A).

**Table 1.** Median Number of Darunavir and Primary PI RAMS Over Time

*All samples*	*Median number of RAMs*		*Samples with phenotypic resistance to one or more PIs*	*Median number of RAMs*	
	*Darunavir*	*Primary PI*		*Darunavir*	*Primary PI*
2010	0	0	2010	1	3
2011	0	0	2011	1	3
2012	0	0	2012	1	3
2013	0	0	2013	1	3
2014	0	0	2014	1	2
2015	0	0	2015	1	3
2016	0	0	2016	1	3
2017	0	0	2017	1	2

PI, protease inhibitor; RAMs, resistance-associated mutations.

**FIG. 3.**
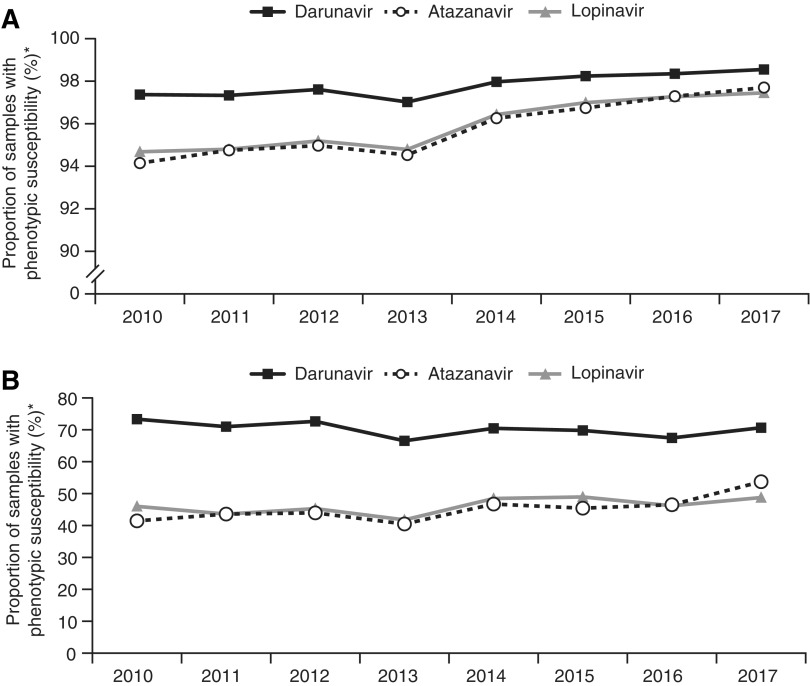
Proportion of samples with phenotypic susceptibility to darunavir, atazanavir, and lopinavir over time among **(A)** all samples and **(B)** samples with phenotypic resistance to one or more PIs.* PI, protease inhibitor; FC-IC_50_, fold change in 50% inhibitory concentration. *Phenotypic resistance was defined by the lower clinical cutoffs for ritonavir-boosted darunavir (FC-IC_50_>10), ritonavir-boosted atazanavir (FC-IC_50_ >5.2), and ritonavir-boosted lopinavir (FC-IC_50_ >9).

The proportion of samples with phenotypic susceptibility to darunavir was 97.4% in 2010 and 98.6% in 2017 (*p* = .013); these values for atazanavir were 94.2% and 97.7% (*p* = .003), respectively, and for lopinavir were 94.7% and 97.5% (*p* = .003; Fig. 3A). The median FC-IC_50_ value for darunavir was 0.76 in 2010 and 0.61 in 2017 (*p* = .009); these values for atazanavir were 0.94 and 0.88 (*p* = .063), respectively, and for lopinavir were 0.82 and 0.74 (*p* = .035; Table 2).

**Table 2.** Median Darunavir, Atazanavir, and Lopinavir FC-IC50 Values Over Time

*All samples*	*Median FC-IC_50_ value*			*Samples with phenotypic resistance to one or more PIs*	*Median FC-IC_50_ value*		
	*Darunavir*	*Atazanavir*	*Lopinavir*		*Darunavir*	*Atazanavir*	*Lopinavir*
2010	0.76	0.94	0.82	2010	2.3	7.1	12
2011	0.77	0.96	0.83	2011	2.5	7.2	13
2012	0.77	0.99	0.83	2012	2.1	6.2	13
2013	0.75	0.95	0.80	2013	2.7	7.7	16
2014	0.68	0.88	0.74	2014	2.3	5.7	9.6
2015	0.62	0.85	0.71	2015	2.0	6.0	9.7
2016	0.60	0.84	0.71	2016	2.5	5.8	13
2017	0.61	0.88	0.74	2017	1.9	4.6	9.6

FC-IC_50_, fold change in 50% inhibitory concentration; PI, protease inhibitor.

### Samples with phenotypic resistance to one or more PIs

The subset of clinical samples with distinct phenotypic resistance to one or more PIs as defined in this study included 4,799 samples, representing 7.9% of all samples. Within this subset, the proportion with 0 darunavir RAMs was 36.4% in 2010 and 42.3% in 2017 (*p* = .216), while the proportion with three or more darunavir RAMs was 27.7% in 2010 and 30.9% in 2017 (*p* = .138; Fig. 1B). Correspondingly, the median number of darunavir RAMs was one for each year from 2010 to 2017 (*p* = 1.000 [2010 vs. 2017]), and the median number of primary PI RAMs was two or three for each year during this time period (*p* = .322 [2010 vs. 2017]; Table 1). The most common individual darunavir RAM was L33F, which was identified in 37.9% of samples in 2010 and 37.8% of samples in 2017 (*p* = .458); the least common darunavir RAM, L76V, was identified in 3.7% of samples in 2010 and 5.0% of samples in 2017 (*p* = .322; Fig. 2B). The relative prevalence of individual darunavir RAMs over time among samples with phenotypic resistance to one or more PIs was similar to that observed across all samples. The most common primary PI RAM, L90 M, was identified in 50.1% of samples in 2010 and 48.3% of samples in 2017 (*p* = .216; Fig. 2B).

The proportion of samples with phenotypic susceptibility to darunavir was 73.3% in 2010 and 70.7% in 2017 (*p* = .138); these values for atazanavir were 41.5% and 53.7% (*p* = .026), respectively, and for lopinavir were 46.0% and 48.8% (*p* = .138; Fig. 3B). The median FC-IC_50_ value for darunavir was 2.3 in 2010 and 1.9 in 2017 (*p* = .322); these values for atazanavir were 7.1 and 4.6 (*p* = .048), respectively, and for lopinavir were 12 and 9.6 (*p* = .386; Table 2).

## Discussion

We previously described a decrease in the prevalence of darunavir RAMs and phenotypic resistance among commercially tested HIV-1 isolates in the US from the time of darunavir approval in 2006 through 2012, stabilizing around 2010.^16^ The expansion of that dataset, which includes a more current sample population, demonstrated that the prevalence of darunavir resistance remained low and generally stable from 2010 to 2017, after the initial observed decrease and despite the increasing use of darunavir relative to other PIs.^32^

As expected, due to the enrichment of samples with protease mutations, darunavir RAMs were more common in the PI-resistant subset of samples compared with all samples. Relative to all samples, higher proportions of PI-resistant samples had three or more darunavir RAMs and lower proportions showed phenotypic susceptibility to darunavir. Notably, the proportion of PI-resistant samples with three or more darunavir RAMs did not significantly change over the 8-year time period. Moreover, the proportion of samples harboring three or more darunavir RAMs and the proportion of samples with reduced phenotypic darunavir susceptibility (Figs. 1 and 3), correspond exactly over time (∼2% among all samples and ∼30% among PI-resistant samples); this correlation has been identified previously and supports a high barrier to resistance for darunavir in which multiple RAMs are needed for loss of virologic suppression.^12,13^ In contrast to darunavir, for other PIs and ARV agents with a lower barrier to resistance, accumulation of relatively fewer RAMs is needed for loss of phenotypic susceptibility.^33^

The high proportion of all samples submitted for routine genotypic/phenotypic resistance testing showing phenotypic susceptibility to darunavir (98.6% in 2017) supports the use of darunavir even if no resistance testing results are available. Moreover, the relatively high proportion of samples with 0 darunavir RAMs among all samples over time (95.8% in 2017), and even among samples with PI resistance (42.3% in 2017), supports the use of darunavir QD in treatment-experienced patients without darunavir RAMs.^8,10,11^

Instances in which an ARV agent with a high barrier to resistance is especially important for initiation of therapy, such as when adherence is a concern or resistance test results are unavailable, are cited in US DHHS guidelines as a clinical scenario in which a darunavir-based regimen is recommended.^1^ In addition to its high barrier to resistance, the high prevalence of darunavir phenotypic susceptibility is another factor to consider in determining treatment strategies for people living with HIV-1 infection, who may be at risk for developing resistance.^1^ Maintenance of virologic suppression may also be aided by improvements in treatment adherence and regimens that combine approaches, such as the use of single-tablet regimens that include ARV agents with a high barrier to resistance.^6,7,24,25^

While the prevalence of darunavir resistance in this analysis was low, it has more rarely been observed in clinical trials. For example, in a recent analysis of seven clinical trials of darunavir 800 mg QD-based regimens, 0.2% of subjects had postbaseline primary PI and/or darunavir RAMs and <0.1% lost phenotypic susceptibility to darunavir.^5^ The observed discrepancy with this study is likely a reflection of the different study populations. Clinical trials enroll patients who meet certain eligibility criteria, which could include restrictions on treatment history, RAMs, and/or phenotypic susceptibility, into a highly controlled study environment. In contrast, in our analyses of the Monogram database,^16^ the clinical samples had been submitted for commercial resistance testing that included combined genotyping and phenotyping. Combination testing is more likely to be utilized, relative to genotyping alone, for individuals who may be expected to have more complicated resistance and a higher degree of resistance.^1^ In the most recent US DHHS guidelines, both genotyping and phenotyping are the preferred options for individuals with known or suspected complex patterns of RAMs.^1^ Also, in these guidelines, boosted darunavir is the preferred boosted PI for individuals with prior treatment failure or demonstrated resistance mutations, as well as for “individuals with uncertain adherence or in whom treatment needs to begin before resistance testing results are available.”^1^ Given this context, a limitation of this analysis is that no ARV treatment history information was available in the Monogram database to assess the prevalence of resistance by treatment experience (i.e., treatment naive vs. treatment experienced).

Notably, the proportion of samples with RAMs or phenotypic resistance in the PI-resistant population was greater in this analysis than reported in our earlier analysis.^16^ This is a consequence of how the subset of samples with phenotypic resistance to one or more PIs was defined, which was more stringent in this analysis. In this study, an FC-IC_50_ of more than two times the lower clinical or biological cutoff for each PI was used, compared with an FC-IC_50_ of more than the lower clinical or biological cutoff in the earlier analysis. A more stringent cutoff was applied to exclude specimens with minimal FC-IC_50_ increases close to the defined cutoff for each of the drugs, which considers the inherent assay reproducibility and results in a population enriched for samples with clear resistance.

In summary, the prevalence of darunavir RAMs among commercially tested HIV-1 samples remained low and generally stable from 2010 to 2017, while high proportions of samples maintained phenotypic darunavir susceptibility. These findings further illustrate the high barrier to resistance of darunavir and, together with recommendations in US DHHS guidelines, suggest that darunavir is a reasonable choice when starting ARV treatment of individuals who may have uncertain adherence and those without baseline resistance testing (such as in a rapid initiation model of care).
